# Injecting Immunosuppressive M2 Macrophages Alleviates the Symptoms of Periodontitis in Mice

**DOI:** 10.3389/fmolb.2020.603817

**Published:** 2020-10-23

**Authors:** Yibin Miao, Liuting He, Xiaoyu Qi, Xiaoping Lin

**Affiliations:** ^1^Department of Stomatology, Shengjing Hospital of China Medical University, Liaoning, China; ^2^Department of Stomatology, The First Affiliated Hospital of Shenzhen University, Shenzhen Second People’s Hospital, Shenzhen, China; ^3^Shenyang Medical College, Liaoning, China

**Keywords:** M2 macrophages, IL-10, inflammation, periodontitis, regulatory T cells

## Abstract

Periodontitis is the second most common oral disease affecting tooth-supporting structures. The tissue damage is mainly initiated by the excessive secretion of proinflammatory cytokines by immune cells. Macrophages are a type of antigen-presenting cells that influence the adaptive immunity function. We used a unique set of cytokines, i.e., a combination of IL-4, IL-13, and IL-10, to stimulate macrophages into a subset of M2 polarization cells that express much higher levels of ARG-1, CD206, and PDL-2 genes. The cells’ anti-inflammatory potential was tested with mixed-lymphocyte reaction assay, which showed that this subset of macrophages could increase IL-2 secretion and suppress IL-17, IL-6, and TNF-α secretion by splenocytes. The gram-negative bacterial species *P*orphyromonas *gingivalis* was used to initiate an inflammatory process in murine periodontal tissues. In the meantime, cell injection therapy was used to dampen the excessive immune reaction and suppress osteoclast differentiation during periodontitis. Maxilla was collected and analyzed for osteoclast formation. The results indicated that mice in the cell injection group exhibited less osteoclast activity within the periodontal ligament region than in the periodontitis group. Moreover, the injection of M2 macrophages sustained the regulatory population ratio. Therefore, the M2 macrophages induced under the stimulation of IL-4, IL-13, and IL-10 combined had tremendous immune modulation ability. Injecting these cells into local periodontal tissue could effectively alleviate the symptom of periodontitis.

## Introduction

Periodontitis is the second most prevalent oral disease, mainly caused by a bacterial infection. Lymphocytes heavily involved in chronic inflammation play a significant role in the disintegration of tooth-supporting structures and contribute to bone resorption, which is the hallmark of periodontitis, followed by tooth loosening. However, the real damage is inflicted not by the bacteria directly, but by the excessive secretion of inflammatory mediators by the local immune cells (Bonner et al., 2018). Periodontitis is an autoimmune disease similar to rheumatoid arthritis. However, the treatment is limited to drugs and the physical removal of dental plaque. Previous research has shown that the effector T cell in the tissue contributes to osteoclast differentiation in the periodontal ligament (Kanzaki et al., 2016). The research about autoimmune diseases have come up with many theories in term of pathogenesis (Cronin et al., 2019), many treatment methods revolve around suppressing the function of infiltrating T cells or enhancing the function of a specific cell type (Chambers and Matosevic, 2019).

Macrophages found in most tissues constitute an essential part of the first defense line against foreign pathogens. There are two major subclasses of macrophages, the proinflammatory M1 subset and anti-inflammatory M2 subtype. In the event of bacterial infection, the macrophages in the tissue quickly polarize into M1 state under the influence of gram-negative bacteria and secrete large amounts of nitric oxide and inflammatory cytokines. On the contrary, when the pathogen is cleared, and the wound begins to heal, the paradigm in the tissue shifts to M2 macrophages, featuring high levels of ARG-1, to promote tissue regeneration and PDL-2 to suppress T cell proliferation (Yu et al., 2016). Regulatory T cells featuring a high expression of Foxp3 play a pivotal role in suppressing the immune system and maintaining tolerance to specific antigens, with its depletion leading to enhanced immunity against pathogens (Sakaguchi, 2003). A deficiency in regulatory T cells also leads to a tendency to develop an allergic reaction. It has been reported that the ratio of regulatory T cells in the progressive state of periodontitis tissue is lower than the healthy tissue (Hays et al., 2019). Macrophages play an essential role in shaping adaptive immunity by presenting antigens and secreting cytokines. The phenotype of macrophages heavily depends on the surrounding microenvironment.

Injecting viable cells into the tissue to induce a medicinal effect has extensively been used to treat many diseases, such as cancer and neurological disorders. The transfer of tumor cell reactive lymphocytes has shown a positive result in the clinical trial (Tran et al., 2008). Stem cells have also been used in the cell transfer therapy to either enhance immunity or promote tolerance (Gattinoni et al., 2012). In the event of chronic inflammation, the immune system regulation became abnormal. In order to regain the control, many immune-suppressive drugs are used to restrain the function of pro-inflammatory cells. The adoptive transfer of anti-inflammatory cells such as the regulatory T cells offers many advantages compared to using drugs (Riley et al., 2009). Previous study has demonstrated that an antigen presentation cell transfer therapy treating pre-induced inflammation. The intra-peritoneal injection of immuno-suppressive macrophages induced higher Foxp3^+^ regulatory T cells ratio in the spleen (Bonnamain et al., 2012). This study transplanted differentiated immune cells instead of undifferentiated progenitor cells. Traditional drug treatment for periodontitis aims to suppress the function of T cells. We hypothesized that the expansion of immune-suppressive cells locally could positively impact the development of inflammatory diseases. The effect of macrophages on T cells has not sufficiently been explored compared to that of T cells on macrophages. This study aimed to investigate the effect of mature immune-suppressive macrophages on T cells *in vitro* and determine how the transplantation of this specific cell subset affects the development of periodontitis *in vivo*.

## Materials and Methods

### Generation of M2 Macrophages

Bone marrow cells were flushed out and passed through a 70-mm nylon mesh into a centrifuge tube. After red blood cells were lyzed, the remaining cells were resuspended and cultured in DMEM medium (Biological Industries, United States) supplement with 10% heat-inactivated fetal bovine serum (Serana, Germany) and 10 ng/mL of M-CSF (Cell Signaling Technology, United States) for several days until all the cells were differentiated into macrophages. The macrophages were equally divided into four groups, including a control group. The second group was stimulated with 10 ng/mL of IL-4 and 10 ng/mL of IL-13 (Sino, China) for 24 h; the third group was treated with 10 ng/mL of IL-10 (Peprotech, United States) for 24 h; the forth group was treated with IL-4, IL-13, and IL-10 for 24 h.

### Real-Time Quantitative Polymerase Chain Reaction (RT-PCR)

After the cells were collected from each group, the total RNA was extracted using Trizol reagent (Invitrogen, United States), and cDNA was synthesized with HiScript II QRT SuperMix with gDNA wiper (Vazyme Biotech, China). SYBR Green-based qPCR was run on a LightCycler 480 (Rouche, United States) using ChamQ Universal SYBR^®^ qPCR Master Mix (Vazyme Biotech, China). The amplification process data were analyzed using Ct (2−ΔΔCt) method.

### Lymphocyte Mixed Culture Method

The lymphocyte mixed culture method was used to explore the immune-suppressive properties of each group of macrophages. Briefly, the splenocytes were extracted from the same donor mice and cultured in the DMEM medium for 12 h to eliminate the remaining macrophages. The splenocytes were then cultured with each macrophage group under a ratio of 10:1 for 5 days. The culture medium was supplemented with 1 mg/mL of CD3 monoclonal antibody (Invitrogen, United States).

### Cell Counting (CCK8) and Cytometric Beads Array (CBA)

The lymphocyte proliferation was determined every day from the first to the fifth day using a CCK8 kit (Vazyme Biotech, China). The optical absorbance was measured at 450 nm by a microplate reader. The cytokines in the culture medium were analyzed using a CBA kit (BD Bioscience, United States); the assay was carried out according to the manufacturer’s instructions. Data were acquired with a BD FACS Calibur flow cytometer and analyzed with the FCAP array software. IL-2, IL-4, IL-6, IFN-g, TNF-a, IL-17, and IL-10 were recorded and compared.

### Animals

Balb/c mice (8–10 weeks old), procured from the China Medical University Laboratory Animal Center, were housed in the laboratory’s standard breeding facility with food and water available *ad libitum*. The mice were divided into three groups: healthy (H), periodontitis (P), and cell injection (CI) groups. Mice in the periodontitis group received *P. gingivalis* injection in the gingival sulcus around the maxillary third molar once every 2 days for three times; mice in the cell injection group received injection of cells suspension once every week. All the procedures were approved by the Animal Etheric Committee of China Medical University.

### *P. gingivalis* Culture and Induction of Periodontitis

*P. gingivalis* was cultured on tryptic soy broth agar in an anaerobic environment. After 7 days, the bacteria colony was collected and suspended in PBS solution, the concentration was adjusted in accordance with the McFarland standards to 10^9^CFU/mL approximately. Equal volume of bacteria suspension and sodium carboxymethyl cellulose were mixed together, a 10 μL mixture was injected into the gingival sulcus of the third molar every 2 days for three times.

### Cell Suspension Injection

The M2 macrophages induced by IL-4, IL-13, and IL-10 were detached using StemPro Accutase (Gibco, United States). The concentration of the cell suspension was adjusted to 10^9^ cells/mL; 10 μL of cell suspension was injected into the gingival sulcus of the third molar through a 33-G needle every week after the last time of *P. gingivalis* injection.

### Flow Cytometry Analysis

The gingival tissue was harvested and analyzed for the cell population. Briefly, the gingival tissue was processed and digested using collagenase at 37°C for 30 min. The cells collected through a nylon mesh were fixed and permeabilized using a Foxp3 staining kit (BD Bioscience, United States). The Foxp3 T cells were marked with perCP-CY5.5 conjugated anti-CD4 antibody (BD Bioscience, United States) and PE-conjugated anti-Foxp3 antibody (Elabscience, China). The M2 macrophage population was marked with PE-conjugated anti-F4/80 antibodies (BD Bioscience, United States) and FITC conjugated anti-CD206 antibody (BD Bioscience, United States). Data were acquired with a BD FACS Calibur flow cytometer.

### Tartrate-Resistant Acid Phosphatase (TRAP) Staining

The c was harvested and fixed in a neutral formalin solution for 48 h to evaluate bone resorption. After fixation, the tissue was subjected to a 7-day decalcification process in a neutral EDTA solution in a warm incubator. The tissues were embedded in the OTC compound and snap-frozen to −80°C; 5-μm cross-sections were obtained and subjected to TRAP staining.

### Statistical Analysis

The results were reported as means ± SDs from three repeated experiments. Statistical analyses were performed with the two-tailed Student’s *t*-test. The regulatory T cell and M2 macrophage population ratios were subjected to the linear regression analysis. *P* < 0.05 was set as the standard for statistical significance.

## Results

### IL-10 Combined With IL-4/IL-13-Activated M2 Macrophages

**FIGURE 1:**
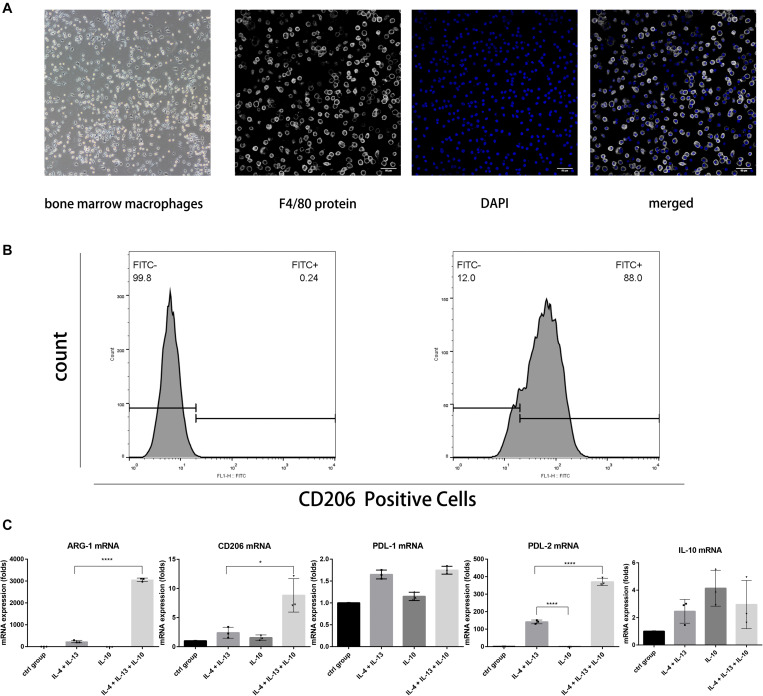
The use of IL-4 and IL-13 with IL-10 in combination to stimulate macrophages resulted in much higher mRNA levels of ARG-1, CD206, and PDL-2 than without them. **(A)** The morphology of bone marrow macrophages and the F4/80 protein immunostaining. The bone marrow cells attached to the bottom and shown macrophage morphology after 5 days culture; the three fluorescent photos are showing F4/80 protein staining with fluorescent antibody, nucleus staining with DAPI and two staining merged together, respectively. **(B)** The flow cytometric analysis of CD206 intracellular staining of macrophages stimulated with IL-4 and IL-13. **(C)** The ARG-1, CD206, PDL-1, PDL-2, and IL-10 mRNA levels of macrophages in each group. The macrophages in the fourth group expressed higher levels of ARG-1, CD206, and PDL-2 mRNA. Data are shown as means ± SD.

Bone marrow cells were collected and stimulated with M-CSF for five consecutive days to obtain and evaluate the mRNA expression of M2 macrophages. The immunofluorescent assay was used to stain the F4/80 membrane protein for its identification (Figure 1A). The results indicated that the bone marrow cells successfully differentiated into macrophages. 5 ng/mL of IL-4 and IL-13 were added to the culture medium to achieve M2 subset differentiation. After 24 h, CD206 expression, the signature protein of M2 macrophages, was analyzed using flow cytometry, as shown in Figure 1B. RT-PCR was used to quantify ARG-1, CD206, and PDL-2 mRNA expression of the macrophages in different groups. The results showed that IL-10 combined with IL-4 and IL-13 results in a much higher ARG-1, CD206, and PDL-2 mRNA levels than IL-4 and IL-13. However, in the third group, macrophages stimulated solely with IL-10 did not exhibit any significant changes. Taken together, using a combination of IL-10, IL-4, and IL-13 to stimulate macrophages could induce a much higher level of M2 anti-inflammatory protein expression (Figure 1C).

### M2 Macrophages Enhance IL-2 Secretion While Reduce IL-6 and IL-7 Secretion in the Mixed Lymphocytes Reaction

**FIGURE 2:**
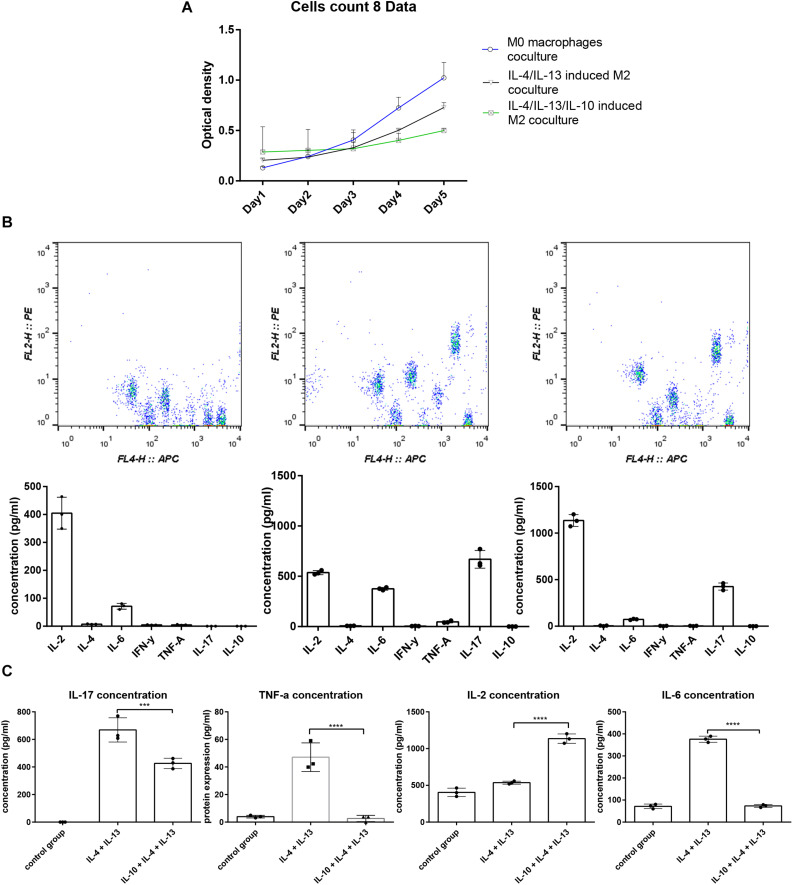
The splenocyte proliferation analysis and cytokine profile difference cocultured with macrophages. **(A)** Five days of splenocyte proliferation in the mixed culture using CCK-8 kit. The relative cell count is represented by optical density (OD) value. **(B)** CBA analysis of cytokine profiles in the supernatant of mixed culture; the IL-4, IL-13, and IL-10 groups exhibited higher IL-2, IL-6, TNF-α, and lower IL-17 concentration compared to the IL-4, IL-13 group.

A mixed cell culture was set up to assess the difference between the three groups of macrophages in terms of immunostimulatory activity. Three groups of macrophages were cultured with splenocytes with the ratio of one macrophage to ten splenocytes (Figure 2A) for 5 days. Daily cell proliferation was analyzed using a CCK8 kit. The results indicated that the macrophages treated with IL-10, IL-4, and IL-13 exhibited a higher ability to suppress lymphocyte proliferation (Figure 2B). Furthermore, the concentration of the cytokines in the culture supernatant was analyzed using a CBA, which showed that the IL-12 level was much higher, and IL-6, IL-17, and TNF-α levels were much lower compared to the macrophages treated by only IL-4 and IL-13 (Figures 2C,D).

### The Injection of M2 Macrophages Increased the Ratio of Regulatory T Cells and Reduced Osteoclast Differentiation

**FIGURE 3:**
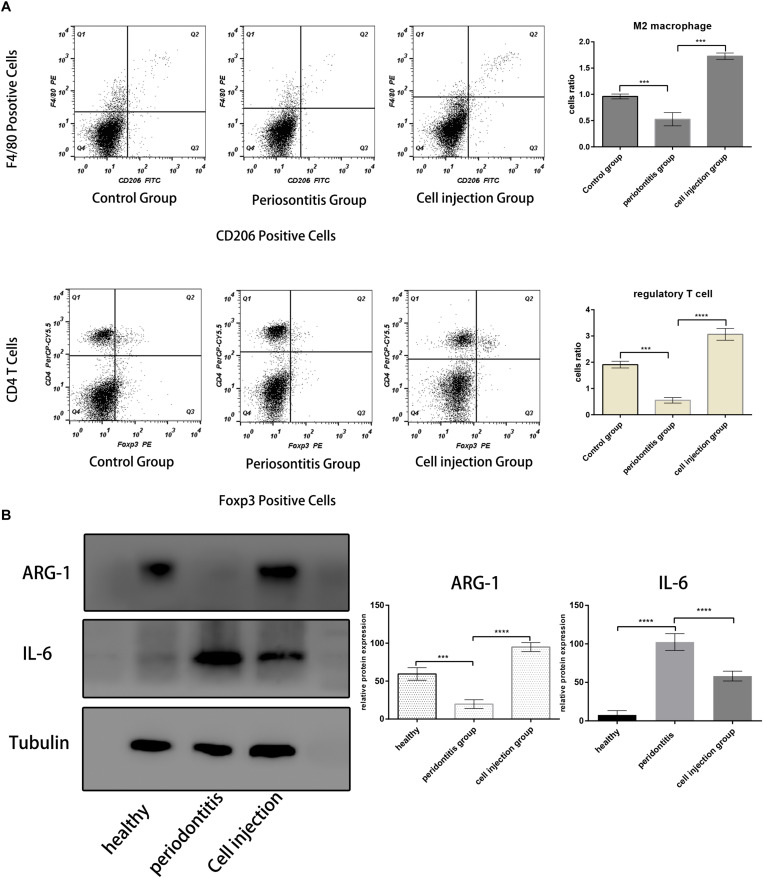
M2 macrophages and regulatory T cell population analysis. **(A,B)** Flow cytometric analysis of cells in the mice periodontal tissue in each group. The M2 macrophages were gated with F4/80^+^ and CD206^+^; the Treg cells were gated with CD4^+^ and Foxp3^+^. Both the ratios of F4/80^+^ and CD206^+^ cells and CD4^+^ and Foxp3^+^ cells are lower in the periodontitis group and higher in the healthy group and cell injection group. **(C)** Western blot analysis of IL-6 and ARG-1 protein in the gingival tissue. The statistical significance was determined by *P*-value (***P < 0.001, ****P < 0.0001).

**FIGURE 4:**
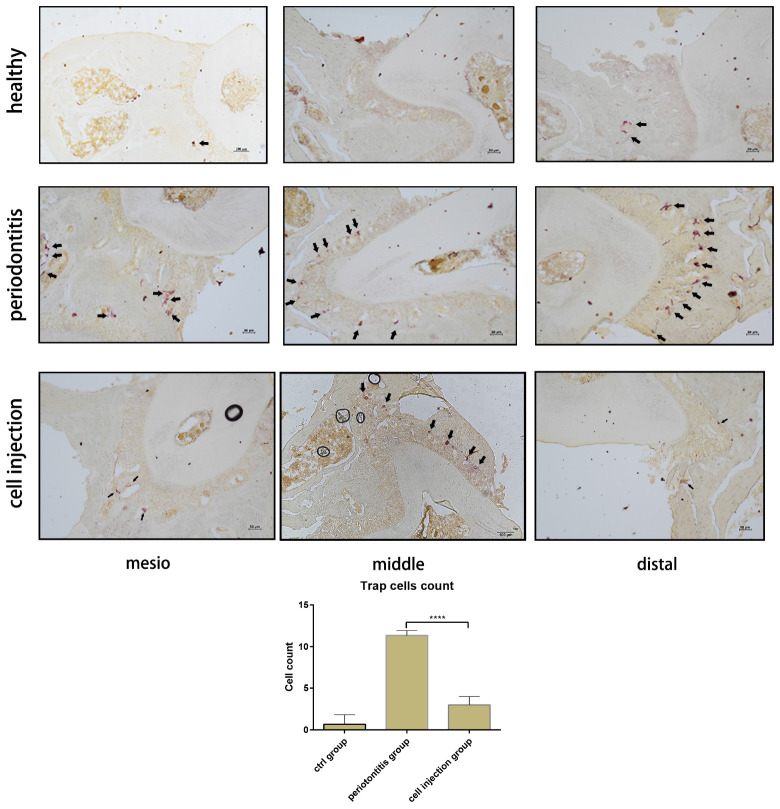
Tartrate-resistant acid phosphatase (TRAP) staining of osteoclast in the periodontal ligament of the distal, middle, and mesial sites of the second molar. The cell counts of TRAP-positive cells are shown as means ± SD; the statistical significance was determined by *P*-value (***P < 0.001).

In the mouse periodontitis model, the ratios of M2 macrophages and regulatory T cells in the gingival tissue were lower than the healthy group, while in the cell injection group, the ratio was higher than the periodontitis group (Figures 3A,B). This finding indicated that the injection of M2 macrophages positively affected the regulatory T cell population. Besides, the ARG-1 protein and proinflammatory cytokine IL-6 levels were determined in the gingiva using western blot. The periodontitis group exhibited an elevated level of IL-6 and a lower level of ARG-1 compared to the healthy group, while in the cell injection group, the IL-6 level was significantly suppressed. TRAP staining showed significant osteoclast differentiation in the periodontal ligament region; however, in the cell injection group, osteoclast activity was significantly low (Figure 4).

## Discussion

The present study compared the immune properties of macrophages activated under various conditions. Stimulating with IL-13 and IL-4 could induce macrophages into an immune-suppressing M2 subtype. Although IL-10 alone could not alter the phenotype of macrophages, when combined with IL-4 and IL-13, the macrophages exhibited a much higher level of M2-related gene expression, capable of promoting IL-2 expression while suppressing IL-6, TNF-α, and IL-17 expression when cultured with splenocytes.

A periodontitis model was induced in mice to monitor the M2 macrophages’ ratios and regulatory T cells in the local gingival tissue using flow cytometry during inflammation. Besides, the mechanism through which the injection of immuno-suppressive macrophages affected the local site of periodontitis was evaluated. The results showed that the ratio of M2 macrophages and regulatory T cells significantly decreased in the periodontitis group’s gingiva compared to the healthy group. This outcome led to the assumption that expanding the M2 macrophage population might positively affect periodontitis treatment. The model in the present study was created with *P. gingivalis* to induce periodontitis in the local region. The infected mice exhibited a decrease in the ratio of M2 macrophages and regulatory T cells compared to the healthy group in the inflamed gingival tissue. Periodontitis is induced mainly by the infection with gram-negative organisms, but the host’s immune response, such as leukocyte activation and osteoclast differentiation, largely contributes to bone resorption (Ernst et al., 2007). Regulatory T cells tend to migrate to the inflammatory tissue as one of the leukocyte populations and balance proinflammatory reactions. However, they are likely to lose their suppressive properties due to a high concentration of cytokines, such as IL-6 and TNF-a, in the bone resorption lesion (Alvarez et al., 2018). In the CI group, the gingival tissue contained a higher level of ARG-1 and a lower level of IL-6 protein concentration. The results indicated that the injection of the immunosuppressive M2 macrophages could expand the M2 macrophage ratio and suppress the cytokine expression of proinflammatory M1 macrophages.

In the present study, the F4/80^+^, CD206^+^, and CD4^+^/Foxp3^+^ cells were monitored in the mice gingival tissue from the healthy (H), periodontitis (P), and cell injection (CI) groups. The M2 macrophages are specialized in wound healing and inflammation resolution (Ferrante and Leibovich, 2012); they express B7 PDL-2 protein as a mechanism to inhibit the expansion of effector T cells while sustaining the regulatory T cell population (DiDomenico et al., 2018). iNOS is the signature of M1 macrophages that can help destroy pathogens; on the contrary, ARG-1 is an M2 macrophage signature that promotes tissue repair (Yang and Ming, 2014). It has been reported that during the progressive state of periodontitis, the M1/M2 ratio is higher than that in the steady-state or healthy tissues (Yang et al., 2018). Compared to the M1 macrophages, the M2 macrophages secrete ornithine from L-arginine via ARG-1 (Mills et al., 2000). Nitric oxide and ornithine exert a different effect; nitric oxide facilities the clearance of pathogens and inhibits cell proliferation, while ornithine promotes tissue repair (Morris, 2007). ARG-1 and iNOS production have been considered as M2 and M1 activities, respectively.

M2 macrophages, induced by a combination of IL-10, IL-4, and IL-13, express much higher levels of ARG-1, PDL-2, and mannose receptor CD206 than regular M2 macrophages induced by IL-4 and IL-13. Although the mannose receptor has little to do with the antigen-presenting process or priming of the immune reaction, the function of CD206 is still to be elucidated (Gazi and Martinez-Pomares, 2009). Macrophages are one of the antigen-presenting cells, and among the first defense cells to encounter pathogens and activate the adaptive immune reaction. The macrophage and splenocyte mixed cultures were used to investigate the effect of different macrophages on the lymphocytes. The quantitative results of cytokine concentrations in the culture supernatant showed that in the normal state, T cells secrete low levels of IL-2 continuously, as shown in the control group, where the splenocytes were cultured with inactivated macrophages. Maintaining a certain level of IL-2 is essential to maintaining immune homeostasis (Arenas-Ramirez et al., 2015).

The splenocytes were also cultured with two groups of M2 macrophages; the cytokine profiles in the culture supernatant showed that the novel M2 macrophages, stimulated by IL-4, IL-13, and IL-10, resulted in a much higher IL-2 production and a lower IL-17, IL-6, and TNF-a production during the culture period compared to traditional M2 macrophages activated by IL-4 and IL-13. The primary source of IL-2 is not regulatory T cells, but the effector T cells; however, the regulatory T cells and their Foxp3 expression mainly depend on IL-2 stimulation (Hill et al., 2007). T cells’ survival and expansion closely relate to level of IL-2 (Polhill et al., 2012). An increase in IL-2 concentration could lead to an increase in the regulatory T cell population, suppressing the effector T cell activation (Amado et al., 2013). The fact that M2 macrophages promote IL-2 while suppressing IL-6, TNF-a, and IL-17 expression indicates great potential in suppressing inflammation.

It is well established that programmed death-ligand protein plays a role in enhancing and sustaining the master transcription factor Foxp3 (Francisco et al., 2009). PD-1 ligand PDL-1 is expressed by many cell types, such as dendritic cells, macrophages, T cells, and B cells. However, the PDL-2 is mainly expressed by dendritic cells and macrophages (Keir et al., 2008). The PD-1 and PDL interaction suppresses effector T cell function and expansion while sustaining the regulatory T cell population by affecting mTOR signaling pathways (Strauss et al., 2007). The cell count in the mixed cell culture indicated that the M2 macrophages induced by IL-10, IL-4, and IL-13 possess a higher suppressive effect on the lymphocyte proliferation than the traditional M2 macrophages.

Macrophages stimulated by a combination of IL-4, IL-13, and IL-10 express significantly higher levels of ARG-1, with possibly more significant potential of immune suppression ability. Immunomodulatory therapy has been employed to treat many autoimmune diseases (Pannell et al., 2016; Kang et al., 2019). However, the problem with regulatory T cells’ adoptive transfer therapy is that T cells are target-specific (Tonkin et al., 2008). The macrophages could influence the function of adaptive immune cells. We developed an immune-suppressive type of M2 macrophages to not only delay the onset of inflammation but also protect tissues from destruction. A change in the M1/M2 ratio is related to the severity of many autoimmune diseases (Fukui et al., 2017). Immunotherapy, such as immunosuppressive drugs, is utilized to suppress the system reaction and increase the Foxp3^+^ cell population (Liu et al., 2015). A study demonstrated that the peritoneal-injected M2 macrophages migrated to the local region and suppressed the activation of infiltrated proinflammatory immune cells (Parsa et al., 2012). This outcome could also relate to the expansion of the regulatory T cell population. In contrast to the study above, our method is to directly deliver macrophages into the local periodontal tissue.

The RANKL protein secreted by effector T cells promotes osteoclast differentiation (Monasterio et al., 2018) and promotes the expression of many osteoclast precursor genes, including TRAP, leading to bone resorption. Previous research has shown that the regulatory T cells suppress osteoclast genesis during periodontitis (Zaiss et al., 2010). We analyzed the M2 macrophage and Foxp3 T cell populations during periodontitis. The results showed that the regulatory T cell ratio in the inflammatory tissue was lower compared to the healthy group. The data from the cell injection group showed that incorporating the M2 macrophage population not only expanded the regulatory T cell population but also effectively reduced the TRAP-positive cell counts. In conclusion, the M2 macrophages induced by IL-10, IL-4, and IL-13 exhibited higher immune-modulating potential than regular M2 macrophages. They were better at suppressing effector T cell proliferation and sustaining the regulatory T cell population. The adoptive transfer of M2 macrophages successfully alleviated the severity of periodontitis.

Moreover, the macrophages could also be used for nano-drug delivery (Xuan et al., 2019). Recently, macrophage-based nanosystems have been employed for periodontitis treatment. Due to their versatile and unique properties, macrophages and macrophage membranes are quite suitable for coating nanoparticles to build biomimetic systems for biomedical applications. Macrophages are emptied through a hypotonic lysis process for obtaining their outer membranes. The drug (artesunate, antibiotics, etc.) loading macrophage membrane surfaces with nanoparticles can be carried out by extrusion or in an ultrasonic bath. Macrophage membrane coatings have been critical in prolonging the circulation time of nano-drug delivery systems *in vivo*, further enhancing the efficiency of drug delivery and therapeutic efficacy (Tong et al., 2016; Zhang et al., 2018; Xia et al., 2020). Therefore, the macrophage-based nanosystems exhibit great potential for controlled drug delivery in periodontitis treatment.

## Data Availability Statement

The raw data supporting the conclusions of this article will be made available by the authors, without undue reservation.

## Ethics Statement

The animal study was reviewed and approved by Animal Etheric Committee of China Medical University.

## Author Contributions

YM conceived and designed the experiments, analyzed the data, and wrote the manuscript. LH and XQ performed the experiments. All authors contributed equally to the article and approved the submitted version.

## Conflict of Interest

The authors declare that the research was conducted in the absence of any commercial or financial relationships that could be construed as a potential conflict of interest.
