# Corrigendum: Injecting Immunosuppressive M2 Macrophages Alleviates the Symptoms of Periodontitis in Mice

**DOI:** 10.3389/fmolb.2022.848875

**Published:** 2022-01-25

**Authors:** Yibin Miao, Liuting He, Xiaoyu Qi, Xiaoping Lin

**Affiliations:** ^1^ Department of Stomatology, Shengjing Hospital of China Medical University, Liaoning, China; ^2^ Department of Stomatology, The First Affiliated Hospital of Shenzhen University, Shenzhen Second People’s Hospital, Shenzhen, China; ^3^ Shenyang Medical College, Shenyang, China

**Keywords:** M2 macrophages, IL-10, inflammation, periodontitis, regulatory T cells

In the original article, there was a mistake in “Figure 4” as published. “The middle picture in the ‘cell injection group’ is misplaced.” The corrected “Figure 4” appears below.

**FIGURE 4:**
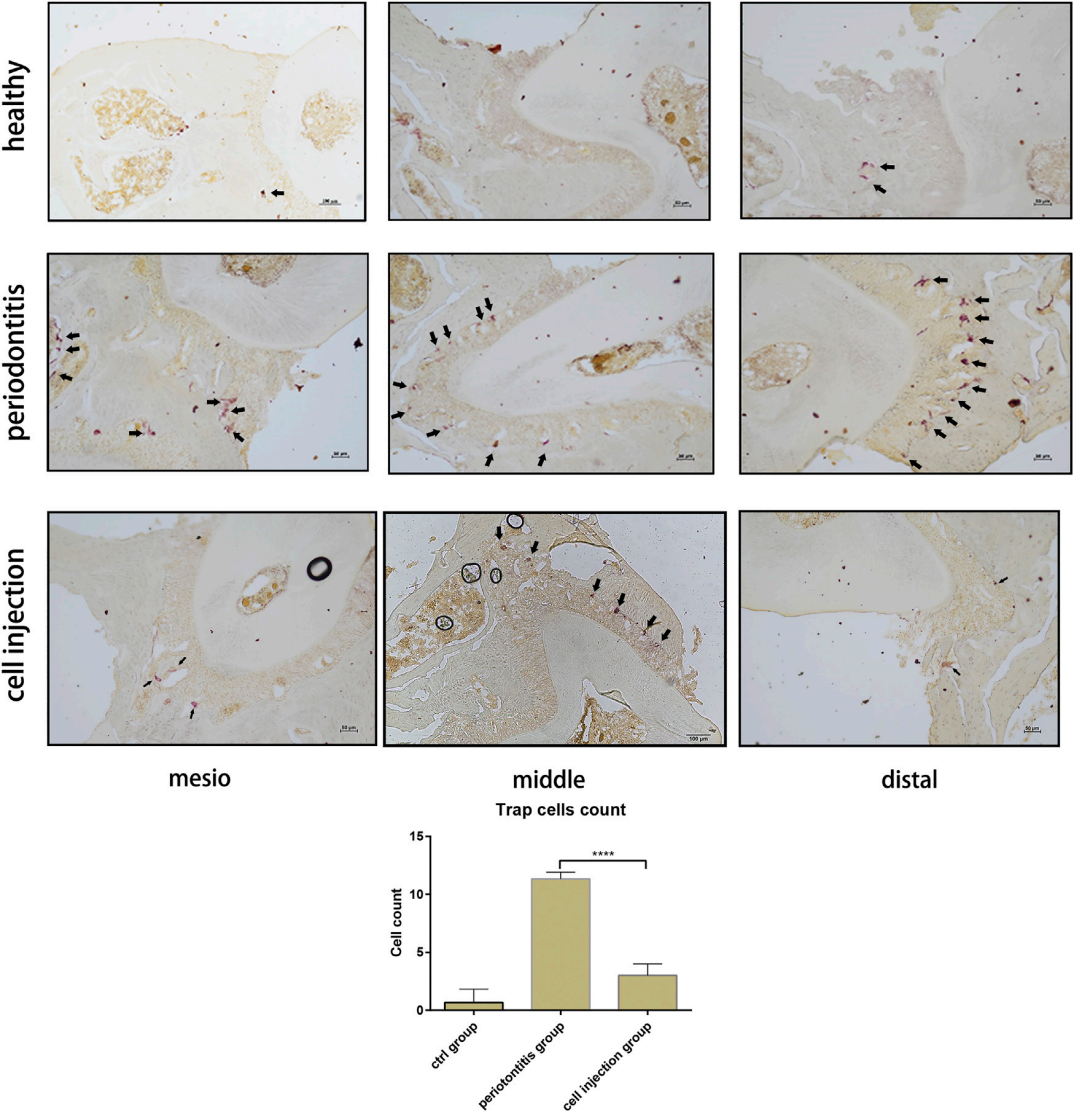
Tartrate-resistant acid phosphatase (TRAP) staining of osteoclast in the periodontal ligament of the distal, middle, and mesial sites of the second molar. The cell counts of TRAP-positive cells are shown as means ± SD; the statistical significance was determined by P-value (***P<0.001).

The authors apologize for this error and state that this does not change the scientific conclusions of the article in any way. The original article has been updated.

