# Supplementary material for: Injecting Immunosuppressive M2 Macrophages Alleviates the Symptoms of Periodontitis in Mice
Source: Front Mol Biosci. 2020 Oct 23;7:603817. doi: 10.3389/fmolb.2020.603817 (PMC7645063; doi:10.3389/fmolb.2020.603817)
Supplement: Supplementary file 1 [file Table_1.DOCX]

***Supplementary Information***

**Injecting Immunosuppressive M2 Macrophages** **Alleviates the Symptoms of Periodontitis in Mice**

**Yibin Miao^1†^, Liuting He^2†^, Xiaoyu Qi^3†^, Xiaoping Lin^1*^**

^1^Department of Periodontology, Shengjing Hospital of China Medical University, 36 Sanhao Street Shenyang, Liaoning 110000, China

^2^Department of Stomatology, the First Affiliated Hospital of Shenzhen University, Shenzhen Second People’s Hospital, 3002 Sungang West Road, Guangdong 518035, China

^3^Shenyang Medical College, 146 Huanghe North Street Shenyang, Liaoning 110000, China

*** Correspondence:**Xiaoping Lin
E-mail: [xiaoping_ba@126.com](mailto:xiaoping_ba@126.com)

†These authors have contributed equally to this work

**Primer sequence for the RT-PCR**

| ARG-1 Forward | CTCCAAGCCAAAGTCCTTAGAG |
| --- | --- |
| ARG-1 Reverse | AGGAGCTGTCATTAGGGACATC |
| PDL-2 Forward | CTGCCGATACTGAACCTGAGC |
| PDL-2 Reverse | GCGGTCAAAATCGCACTCC |
| GAPDH Forward | AGGTCGGTGTGAACGGATTTG |
| GAPDH Reverse | TGTAGACCATGTAGTTGAGGTCA |
| IL-10 Forward | GCTCTTACTGACTGGCATGAG |
| IL-10 Reverse | CGCAGCTCTAGGAGCATGTG |
| PDL-1 Forward | GCTCCAAAGGACTTGTACGTG |
| PDL-1 Reverse | TGATCTGAAGGGCAGCATTTC |
| CD206 Forward | CTCTGTTCAGCTATTGGACGC |
| CD206 Reverse | CGGAATTTCTGGGATTCAGCTTC |
